# Perceptions, Attitudes, and Challenges: Are Tunisian Medical Residents Prepared to Address Gender-Based Violence?

**DOI:** 10.1192/j.eurpsy.2025.2393

**Published:** 2025-08-26

**Authors:** K. Mahfoudh, A. Hakiri, A. Jaoua, I. Besbes, A. Bouallagui, G. Amri, R. Ghachem

**Affiliations:** 1 Department B, Razi Hospital, Manouba, Tunisia

## Abstract

**Introduction:**

Violence against women is a worldwide critical public health issue. Despite the introduction of legal frameworks in Tunisia, there remains a need for effective medical intervention and support. Tunisian Medical residents often serve as frontline responders to cases of violence, making their training and awareness crucial in addressing this public health concern.

**Objectives:**

This study aimed to evaluate the knowledge and attitudes of medical residents in Tunisia concerning the management of gender based violence (GBV).

**Methods:**

A cross-sectional survey was conducted using an online questionnaire distributed via social media, completed by 85 medical residents from various specialties. The study included residents from psychiatry, family medicine, medical, and surgical specialties, providing a comprehensive overview of perceptions across disciplines.

**Results:**

We included 85 medical residents. The preliminary results showed that 73% were aged 26 to 29 years, and 75% were women.

Specialties included 38.8% in psychiatry or child psychiatry, followed by family medicine (25.9%), medical specialties (24.7%), surgical specialties (3.5%), and medico-surgical (7.1%).

Only 21.2% of residents had received specific training on managing GBV during their studies ( Courses, Masterclass, brief training). Regarding prevalence, only 12.9% of participants believed that over 80% of Tunisian women had experienced violence at least once in their lifetime.

In terms of violence types, 35.5% and 17.6% were respectively unaware of economic and political violence.

For 60% of residents, psychological violence was seen as the most prevalent in Tunisia, followed by physical violence (25.9%) and significantly underestimated sexual violence (8.2%). Additionally, 78.8% identified intimate partners as the primary perpetrators of violence. Regarding reporting procedures for suspected gender-based violence cases, 60% of residents believed local authorities should be alerted. However, 48.2% were unaware of Organic Law No. 58 of August 11, 2017, concerning the elimination of violence against women.

Regarding management, only 7.1% of residents felt confident in their ability to handle cases of GBV. The main obstacles identified were a lack of specific training (84.7%), absence of institutional support (67.1%), and lack of time (18.8%). The need for ongoing training in managing GBV was expressed by 64.7% of residents, highlighting the urgent need for improved awareness and skill enhancement in this area.

**Conclusions:**

This study reveals significant gaps in the training and knowledge of medical residents in Tunisia regarding GBV. The lack of awareness of relevant legislation and resources, along with insecurity in case management, underscores the urgent need for targeted training and institutional support to improve medical responses to this critical issue.

**Disclosure of Interest:**

None Declared

